# Prognostic role and relationship of thyroid dysfunction and lipid profile in hospitalized heart failure patients

**DOI:** 10.1002/clc.24057

**Published:** 2023-05-25

**Authors:** Ping Zhou, Liyan Huang, Mei Zhai, Yan Huang, Xiaofeng Zhuang, Huihui Liu, Yuhui Zhang, Jian Zhang

**Affiliations:** ^1^ Heart Failure Center, State Key Laboratory of Cardiovascular Disease, Fuwai Hospital, National Center for Cardiovascular Diseases, Chinese Academy of Medical Sciences and Peking Union Medical College Beijing China

**Keywords:** dyslipidemia, heart failure, lipid, thyroid function, triiodothyronine

## Abstract

**Background:**

Thyroid dysfunction might have a negative impact on the prognosis of patients with heart failure (HF) and affect the lipid metabolism. The aim of our study was to investigate the prognostic role of thyroid dysfunction and its relationship with lipid profile in hospitalized HF patients.

**Hypothesis:**

Thyroid dysfunction strongly correlates with prognosis of HF patients and combination with lipid profile improves the prognostic value.

**Methods:**

We performed a single‐center retrospective cohort study including hospitalized HF patients between March 2009 and June 2018.

**Results:**

Among enrolled 3733 patients, low fT3 (hazard ratio [HR] 1.33; 95% CI: 1.15–1.54; *p* < .001), elevated TSH (HR 1.37; 95% CI 1.15–1.64; *p* < .001), LT3S (HR 1.39; 95% CI: 1.15–1.68; *p* < .001), overt hyperthyroidism (HR 1.73; 95%CI: 1.00–2.98; *p* = .048), subclinical hypothyroidism (HR 1.43; 95%CI: 1.13–1.82; *p* = .003) and overt hypothyroidism (HR 1.76; 95%CI: 1.33–2.34; *p* < .001) independently increased the risk of composite endpoint defined as the combination of all‐cause mortality, heart transplantation, or left ventricular assist device requirement. Higher total cholesterol (HR 0.64; 95%CI: 0.49–0.83; *p* < .001) was still a protective factor in HF patients. When divided into four groups by fT3 and median lipid profiles, comparison of Kaplan–Meier survival curves for various groups showed good risk stratification (*p* < .001).

**Conclusion:**

LT3S, overt hyperthyroidism, subclinical and overt hypothyroidism were independently associated with poor outcomes in HF. The combination of fT3 and lipid profile improved the prognostic value.

## Graphical abstract

The study performed a large single‐center retrospective cohort study to investigate the prognostic role of thyroid dysfunction and the relationship with lipid profile in hospitalized heart failure (HF) patients. Among enrolled 3733 patients, low fT3 and elevated TSH independently increased the risk of composite endpoint defined as the combination of all‐cause mortality, heart transplantation, or left ventricular assist device requirement. Higher total cholesterol was still a protective factor in HF patients and showed good risk stratification combined with fT3 in Kaplan–Meier survival curves.

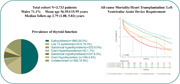

## 1 INTRODUCTION

Thyroid hormones (THs) have several relevant effects on cardiac function by affecting cardiac electrophysiology and contractility, vascular resistance, and even lipid metabolism. Therefore, the occurrence of thyroid dysfunction can favor the onset and progression of several cardiovascular diseases, such as atrial or ventricular arrhythmia, dyslipidemia, atherosclerosis, hypertension, and heart failure (HF), determining a worse prognosis in terms of hospitalization, heart transplantation, and even mortality.^1^

Multiple cohort studies have examined the relationship between thyroid function, and incident atrial fibrillation, HF, and coronary heart disease. In particular, subclinical hypothyroidism and hyperthyroidism were known to be associated with worsening left ventricular systolic and diastolic function, heart rate, and systemic vascular resistance, especially when thyroid‐stimulating hormone (TSH) > 10 mLU/L or <0.1 mLU/L.^2^, ^3^ Furthermore, low triiodothyronine (T3) syndrome (LT3S), defined as the presence of low T3 levels with normal levels of TSH and normal free thyroxine (fT4) levels, is associated with worse outcomes in higher mortality among hospitalized HF,^4^ both in patients with acute HF^5^ and chronic HF.^6^

THs have multiple effects on lipid metabolism, including synthesis, mobilization, and degradation.^7^ It has been gradually recognized that thyroid dysfunction, including overt or subclinical hypothyroidism, is associated with higher total cholesterol (TC), low density‐lipoprotein cholesterol (LDL‐C) levels and triglyceride (TG). Changes in these lipid parameters can lead to progressive lipid accumulation, plaque formation in the arteries, and deleterious effects on cardiovascular disease (CVD), the leading cause of death worldwide.^8^ However, it seems that low LDL‐C levels may predict a less favorable outcome in advanced HF.^9^ The link between comprehensive lipid profile and thyroid function in HF is uncertain.

With complete information on thyroid function in a large cohort of HF patients, we aimed to investigate the prevalence and prognostic effect of thyroid hormone abnormality in hospitalized patients and its relationship with lipid metabolism.

## 2 METHODS

### 2.1 Study population

We enrolled 5124 patients hospitalized in Heart Failure Care Unit (HFCU), Fuwai Hospital, for symptomatic HF from March 2009 to June 2018. The diagnosis of HF was made by two independent cardiologists based on guidelines.^10^ After excluding patients (a) whose thyroid function tests were absent (1289 patients); (b) combined with systemic diseases (102 patients) such as amyloidosis, autoimmune diseases, or active malignant tumors, 3733 patients were included in the final analysis. Clinical information was collected, including medical history, vital signs, laboratory tests, and main treatments. Written informed consent was obtained from the participants, and the study was approved by the local ethics committee of Fuwai Hospital.

### 2.2 Measurement of thyroid function

The thyroid function measurement was evaluated at admission to HFCU. Twelve‐hour fasting blood samples were drawn, and serum‐free T3 (fT3), fT4, total T3 (TT3), total T4 (TT4), and TSH were measured by using radioimmunoassay (Immulite 2000; Siemens, Germany) in the Nuclear Medicine Department of Fuwai Hospital. The reference ranges for fT3, fT4, and TSH were 2.3–4.2 pg/mL, 0.89–1.76 ng/dL, and 0.55–4.78 mLU/L, respectively. Patients were divided according to thyroid function tests as follows^11^: euthyroidism defined as normal TSH, fT3, and fT4. LT3S (fT3 < 2.3 pg/mL with TSH and fT4 within the reference range), subclinical hyperthyroidism (TSH < 0.55 mLU/L with fT3 and fT4 within the reference range), subclinical hypothyroidism (TSH > 4.78 mIU/L with fT3 and fT4 within the reference range), overt hyperthyroidism (TSH < 0.55 mLU/L with elevated fT3 and/or fT4), overt hypothyroidism (TSH > 4.78 mLU/L with decreased fT3 and/or fT4), those who did not fulfill these definitions were assigned to “undetermined.”

### 2.3 Follow‐up and endpoint

Follow‐up data were obtained by reviewing the patients’ hospital records, interviewing the patients via telephone, and examining the outpatient record. The composite endpoint was defined as the combination of all‐cause mortality, heart transplantation, or left ventricular assist device requirement. The data was primarily obtained from death certificates, post‐mortem reports, and medical records. Death caused by accidents was excluded. The median follow‐up duration was 2.79 (1.00, 5.03) years.

### 2.4 Statistical analysis

Descriptive statistics were used to examine the baseline characteristics of the study population at baseline. Data were described as the mean ± standard deviation for normally distributed continuous parameters, as the median (interquartile range) for skewed distributed variables, and frequencies (percentages) for categorical variables. Continuous normally distributed variables were tested with the Student's *t*‐test, skewed variables were tested with the Mann–Whitney *U* test, and categorical variables were tested with chi‐squared tests as appropriate. Bonferroni's correction was performed in multiple comparisons between thyroid dysfunction and euthyroidism groups, and a *p* < .01 was considered statistically significant. LDL‐C levels of the whole population were grouped into quartiles. We also grouped LDL‐C, HDL‐C, TC, and TG levels of the whole population into two groups based on median value. Cox proportional hazards models were performed to assess the association between thyroid dysfunction or lipid profile and risk of death. Model 1 indicated unadjusted; Model 2 was adjusted for age, sex, and baseline BMI; Model 3 was further adjusted for systolic blood pressure (SBP), heart rate (HR), serum sodium, total bilirubin (TBiL), alanine transaminase (ALT), TC, creatinine, log transformed N‐terminal pro‐B‐type natriuretic peptide (NT‐proBNP), left ventricular ejection fraction (LVEF), New York Heart Association (NYHA) functional class, ischemic causes, comorbidities of hypertension, diabetes or atrial fibrillation (AF), medication of β‐blocker, angiotensin‐converting enzyme inhibitor (ACEI), angiotensin receptor antagonist (ARB), and aldosterone receptor antagonist (MRA). In addition, we conducted a subgroup analysis to assess the potential heterogeneity of association between low T3 and composite endpoint. Interactions between low T3 and relevant variables were estimated by means of Cox proportional hazard analysis. Kaplan–Meier analysis was used to assess the cumulative survival of fT3, TSH, and lipid profiles. Furthermore, the association between the level of thyroid hormones and the endpoint was evaluated on a continuous scale with a restricted cubic spline curve based on Cox proportional hazards models adjusted by Model 2. The association between thyroid function and lipid profile was determined using Spearman's correlation analysis and Kaplan–Meier analysis. Two‐tailed *p* values < .05 were assumed as statistical significance. All analyses were performed using R 3.6.2 (R Foundation for Statistical Computing).

## 3 RESULTS

### 3.1 Baseline characteristics of the study population

The included 3733 patients were divided into the following subgroups based on thyroid status: euthyroidism, LT3S, subclinical hypothyroidism, overt hypothyroidism, subclinical hyperthyroidism, overt hyperthyroidism, and undetermined. The most frequent thyroid dysfunction was LT3S (16.3%), followed by subclinical hypothyroidism (6.3%), subclinical hyperthyroidism (6.0%), overt hypothyroidism (4.4%), and overt hyperthyroidism (1.1%). The baseline characteristics of patients in each group are shown in Table 1. Significant differences were seen in BMI, systolic blood pressure, NT‐proBNP, high‐sensitivity C‐reactive Protein (hs‐CRP), NYHA class, AF, and lipid profile of patients with different thyroid status. Notably, LT3S, overt hyperthyroidism, and overt hypothyroidism were associated with lower serum lipid concentrations such as TC, TG, high‐density lipoprotein cholesterol (HDL‐C) or LDL‐C in HF patients.

**Table 1:** Demographic characteristics of the patients in the study population.

Variables	Total (*N* = 3733)	Euthyroidism (*N* = 1865)	Low T3 syndrome (*N* = 610)	Subclinical hyperthyroidism (*N* = 223)	Overt hyperthyroidism (*N* = 42)	Subclinical hypothyroidism (*N* = 237)	Overt hypothyroidism (*N* = 164)
Male	2655 (71.1)	1422 (76.2)	401 (65.7)*	160 (71.7)	21 (50.0)*	151 (63.7)	90 (54.9)*
Age	56.95 ± 15.99	55.24(15.41)	60.48 ± 15.99*	58.62 ± 15.88*	57.33 ± 13.77	54.72 ± 16.89	59.87 (16.73)*
BMI (kg/m^2^)	24.22 [21.61, 27.12]	24.62 [22.21, 27.55]	23.24 [20.45, 26.03]*	23.54 [21.78, 26.42]*	22.50 [20.73, 26.19]*	24.82 [21.77, 28.16]	23.44 [20.53, 27.23]*
Heart rate（bpm）	78 [67, 90]	77 [68, 89]	80 [68, 91]	78 [68, 88]	77 [69, 95]	78 [66, 92]	73 [63, 85]*
NYHA Class III or IV	2386 (71.2)	1103 (64.6)	475 (88.3)*	152 (77.6)*	30 (81.1)	145 (65.3)	123 (82.6)*
Systolic blood pressure (mmHg)	119.35 ± 20.35	122.10 ± 19.56	113.80 ± 21.77*	115.81 ± 20.83*	110.74 ± 18.53*	119.34 ± 16.94	117.90 ± 20.11*
LVDD (mm)	60.77 ± 12.53	61.26 ± 12.06	61.11 ± 13.40	61.72 ± 12.69	61.00 ± 15.24	61.22 ± 12.24	58.30 ± 12.73*
LVEF (%)	41.23 ± 14.73	41.31 ± 14.51	40.00 ± 15.09	40.24 ± 14.71	37.88 ± 12.85	41.52 ± 15.27	43.60 ± 16.00
Laboratory values							
Hemoglobin (g/L)	136.72 ± 22.88	140.61 ± 20.63	129.37 ± 25.11*	135.71 ± 23.03*	134.07 ± 26.30	140.36 ± 22.29	127.40 ± 25.47*
Albumin (g/L)	39.49 ± 5.30	40.65 ± 4.74	36.54 ± 5.36*	39.87 ± 4.84	39.21 ± 4.57	40.83 ± 5.06	37.56 ± 5.91*
ALT (U/L)	22.00 [14.00, 38.00]	23.00 [14.00, 36.00]	21.00 [13.25, 42.00]	23.00 [14.00, 41.00]	23.00 [15.00, 41.25]	23.00 [17.00, 39.00]	18.00 [11.00, 35.00]*
TBiL (μmol/L)	20.10 [13.90, 30.50]	18.80 [13.40, 27.22]	25.35 [16.00, 44.82]*	18.70 [13.45, 25.85]	27.00 [19.85, 43.95]*	20.30 [14.30, 30.10]	22.52 [14.62, 39.01]*
Na (mmol/L)	137.77 [135.00, 140.00]	138.41 [136.00, 140.50]	135.70 [132.30, 138.70]*	138.00 [135.00, 140.26]	137.00 [133.07, 140.59]	137.48 [134.17, 140.00]*	136.10 [134.00, 139.21]*
Creatinine (μmol/L)	92.30 [76.75, 114.20]	87.80 [75.57, 106.25]	105.02 [83.62, 137.02]*	93.07 [79.95, 110.71]*	88.40 [73.00, 120.41]	95.03 [75.50, 116.11]	98.68 [80.80, 124.86]*
HbAlc (%)	6.30 [5.80, 7.00]	6.20 [5.70, 7.00]	6.40 [6.00, 7.20]*	6.20 [5.80, 7.10]	6.30 [5.80, 6.70]	6.30 [5.80, 7.00]	6.20 [5.80, 6.90]
hsCRP (mg/L)	3.93 [1.68, 10.69]	2.84 [1.35, 7.30]	9.30 [3.42, 12.57]*	4.13 [1.72, 10.49]*	4.93 [1.71, 12.25]	3.04 [1.51, 7.41]	6.22 [2.77, 11.64]*
cTnI (ng/mL)	0.02 [0.01, 0.06]	0.01 [0.00, 0.05]	0.02 [0.01, 0.08]	0.02 [0.01, 0.13]*	0.02 [0.01, 0.04]	0.01 [0.01, 0.03]	0.02 [0.01, 0.07]
NT‐proBNP (pg/mL)	1974 [803, 4589]	1389 [601, 3238]	4102 [1779, 8481]*	2114 [986, 4343]*	2603 [1499, 5770]*	2130 [916, 4190]*	2902 [1445, 6541]*
Lipid profile							
TG (mmol/L)	1.32 [0.98, 1.83]	1.41 [1.05, 1.95]	1.14 [0.86, 1.54]*	1.34 [0.99, 1.73]	1.02 [0.79, 1.35]*	1.43 [1.02, 2.05]	1.14 [0.87, 1.53]*
TC (mmol/L)	4.01 [3.31, 4.80]	4.15 [3.45, 4.91]	3.61 [3.02, 4.36]*	4.10 [3.45, 4.73]	3.62 [2.44, 4.57]*	4.07 [3.34, 4.96]	3.68 [2.97, 4.40]*
HDL‐C (mmo/L)	0.95 [0.78, 1.18]	0.97 [0.82, 1.18]	0.87 [0.68, 1.10]*	0.97 [0.84, 1.19]	0.90 [0.72, 1.12]	0.98 [0.81, 1.19]	0.90 [0.70, 1.24]
LDL‐C (mmol/L)	2.41 [1.89, 3.03]	2.52 [2.00, 3.11]	2.19 [1.73, 2.76]*	2.39 [1.98, 3.01]	2.19 [1.32, 2.94]*	2.44 [1.88, 3.06]	2.21 [1.71, 2.87]*
Implantable cardioventer defibrillator	76 (2.0)	27 (1.4)	17 (2.8)	3 (1.3)	4 (9.5)*	7 (3.0)	10 (6.1)*
Ischemic cause of heart failure	1439 (38.5)	738 (39.6)	225 (36.9)	92 (41.3)	10 (23.8)	75 (31.6)	53 (32.3)
Comorbidity							
Hypertension	1821 (48.8)	943 (50.6)	267 (43.8)*	106 (47.5)	15 (35.7)	111 (46.8)	80 (48.8)
Diabetes	1087 (29.1)	518 (27.8)	194 (31.8)	76 (34.1)	11 (26.2)	65 (27.4)	45 (27.4)
Atrial fibrillation	1183 (31.7)	681 (36.5)	259 (42.5)*	60 (26.9)	19 (45.2)*	85 (35.9)*	79 (48.2)*
Medication							
ACEI/ARB	2064 (57.4)	1199 (65.7)	232 (40.5)*	116 (53.2)*	18 (45.0)	138 (59.7)	68 (43.6)*
β‐blocker	3120 (86.8)	1624 (88.9)	472 (82.4)*	191 (87.6)	37 (92.5)	203 (87.9)	121 (77.6)*
MRA	2463 (68.5)	1270 (69.6)	394 (68.8)	134 (61.5)	28 (70.0)	167 (72.3)	107 (68.6)
Diuretics	3139 (87.6)	1560 (87.2)	512 (87.7)	193 (88.9)	37 (92.5)	197 (87.9)	143 (89.4)
Digoxin	1785 (49.7)	898 (49.2)	313 (54.6)	116 (53.2)	20 (50.0)	95 (41.1)*	72 (46.2)

*Note*: Values are presented as mean ± SD or *n* (%). Median (25th‐75th percentile) shown and Wilcoxon rank‐sum test performed because these variables were non‐normal distribution.

Abbreviations: ACEI, angiotensin‐converting enzyme inhibitor; ALT, aspartate transaminase; ARB, angiotensin receptor blocker; BMI, body mass index; cTnI, cardiac troponin I; HbAlc, glycosylated hemoglobin; HDL‐C, high‐density lipoprotein cholesterol; hsCRP, high‐sensitivity C‐reactive protein; LDL‐C, low density‐lipoprotein cholesterol; LVDD, left ventricular end‐diastolic dimension; LVEF, left ventricular ejection fraction; NT‐proBNP, N‐terminal pro‐B‐type natriuretic peptides; NYHA, New York Heart Association; TBiL, total bilirubin; TC, total cholesterol; TG, triglyceride.

* *p* < .01 was considered significant for comparison between thyroid dysfunction and euthyroidism group after Bonferroni's correction.

### 3.2 Survival analysis based on thyroid hormones and thyroid function

**Figure 1:**
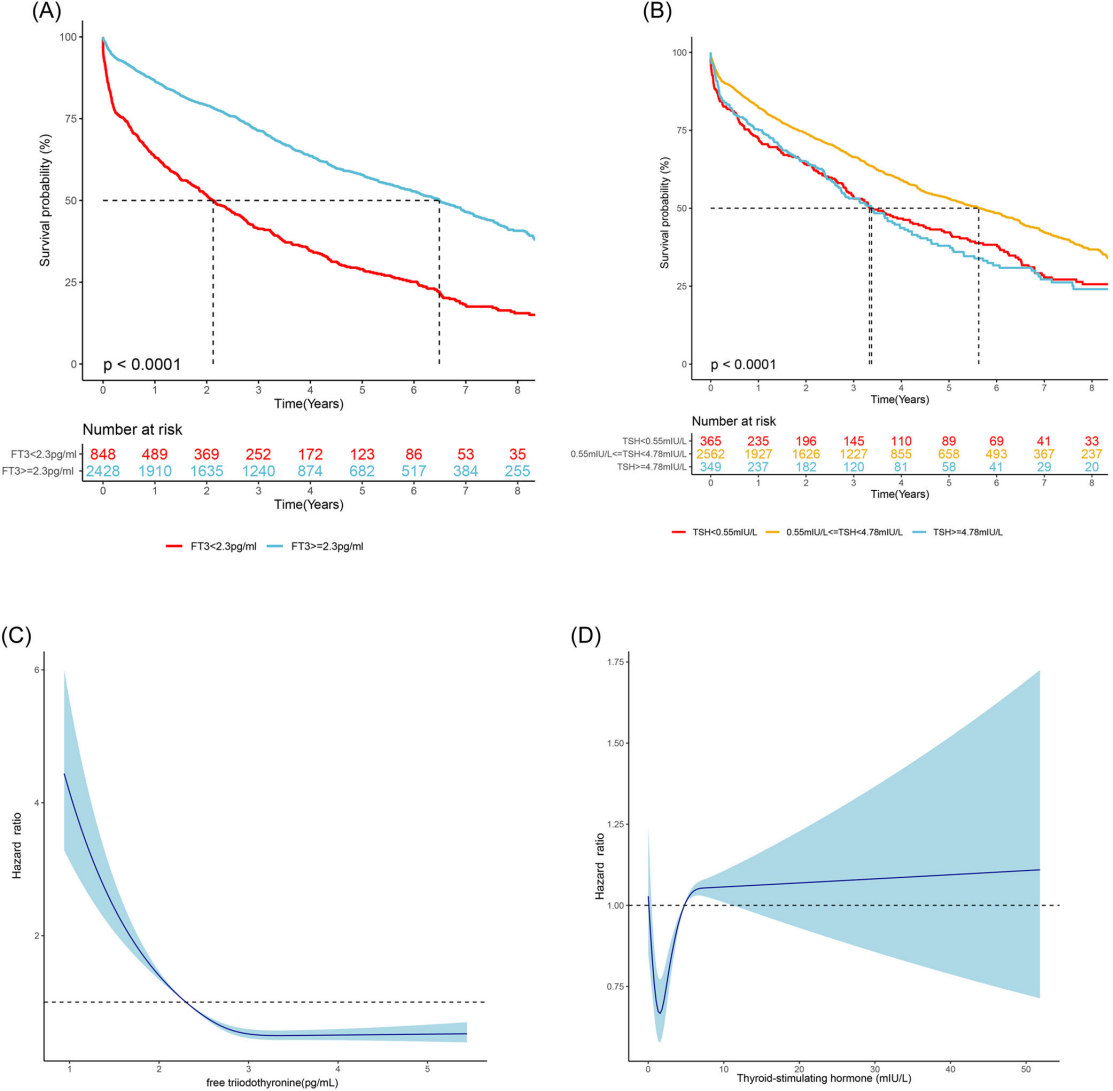
Kaplan–Meier survival curves (A and B) and regression spline based on curve (C and D) with multivariate models (adjusted for age, sex, and BMI) to investigate the relation of serum thyroid hormones and composite endpoint. BMI, body mass index.

**Figure 2:**
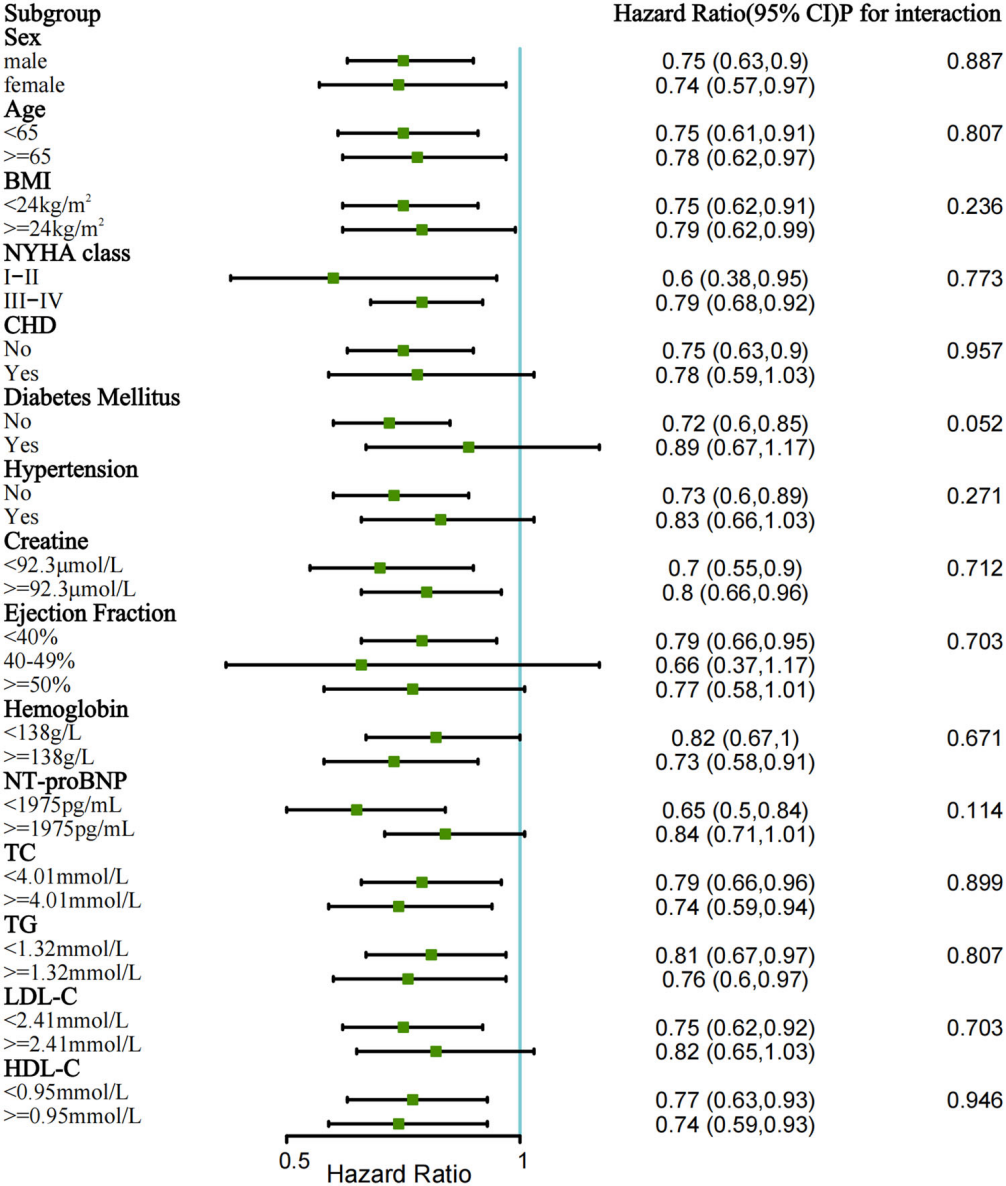
Low fT3 for the prediction of composite endpoint: subgroup analysis. BMI, body mass index; CHD, coronary heart disease; CI, confidence interval; HDL‐C, high‐density lipoprotein cholesterol; LDL‐C, low density‐lipoprotein cholesterol; NT‐proBNP, N‐terminal pro‐B‐type natriuretic peptides; NYHA, New York Heart Association; TC, total cholesterol; TG, triglyceride.

Univariate and multivariate Cox regression analysis of thyroid hormones, thyroid function, and composite endpoint are shown in Table 2. The univariate model showed that low fT3 was a strong predictor of composite outcome (HR 2.43; 95% CI: 2.17–2.70; *p* < .001), as well as abnormal TSH (low TSH: HR 1.41; 95%CI: 1.22–1.63; elevated TSH: HR 1.47; 95% CI: 1.26–1.71; *p* < .001). In multivariate analysis, low fT3 (HR 1.33; 95%CI: 1.15–1.54; *p* < .001) and elevated TSH (HR 1.37; 95%CI 1.15–1.64; *p* < .001) were still independent predictors of worse outcome even after adjustment for the maximum number of variables of Model 3. Kaplan–Meier survival curves about thyroid hormones were shown in Figure 1, and significant differences in composite outcomes were observed in subgroups divided by the level of fT3 or TSH (*p* < .001). These results were corroborated with regression splines that demonstrated a linear trend for risk of composite outcome across fT3 and a U‐shape trend for death risk across TSH (Figure 1). The wide tail of the line was attributed to the limited samples with higher TSH, especially over 10 mLU/L. As for thyroid function, LT3S (HR 1.39; 95%CI: 1.15–1.68; *p* < .001), overt hyperthyroidism (HR 1.73; 95%CI: 1.00–2.98; *p* = .048), subclinical hypothyroidism (HR 1.43; 95%CI: 1.13–1.82; *p* = .003) and overt hypothyroidism (HR 1.76; 95%CI: 1.33–2.34; *p* < .001) were all independent risks of composite endpoint adjusted for Model 3. In the subgroup analysis, lower fT3 was independently associated with composite endpoint across all patient subgroups, including lipid subgroups (Figure 2).

**Table 2:** Univariate and multivariate Cox proportional hazards models for composite endpoint mortality according thyroid function and lipid profile.

Variables	Model 1		Model 2		Model 3	
	Hazard ratio (95% CI)	*p* value	Hazard ratio (95% CI)	*p* value	Hazard ratio (95% CI)	*p* value
Thyroid hormones						
Low fT3	2.43 (2.17–2.70)	<.001	2.33 (2.04–2.56)	<.001	1.33 (1.15–1.54)	<.001
Low fT4	1.08 (0.86–1.33)	.53	1.01 (0.77–1.28)	.94	1.11 (0.83–1.47)	.494
Normal TSH						
Low TSH	1.41 (1.22–1.63）	<.001	1.24 (1.05–1.45)	.009	1.08 (0.89–1.3)	.45
Elevated TSH	1.47 (1.26–1.71)	<.001	1.50 (1.28–1.75)	<.001	1.37 (1.15–1.64)	<.001
Thyroid function						
Low T3 syndrome	2.59 (2.28–2.95)	<.001	2.58 (2.25–2.97)	<.001	1.39 (1.15–1.68)	<.001
Subclinical hyperthyroidism	1.36 (1.1–1.68)	.004	1.33 (1.06–1.65)	.012	1.14 (0.87–1.48)	.344
Overt hyperthyroidism	2.31 (1.49–3.56)	<.001	2.17 (1.38–3.4)	<.001	1.73 (1.00–2.98)	.048
Subclinical hypothyroidism	1.63 (1.32–2.00)	<.001	1.67 (1.35–2.06)	<.001	1.43 (1.13–1.82)	.003
Overt hypothyroidism	2.49 (2.01–3.10)	<.001	2.48 (1.96–3.15)	<.001	1.76 (1.33–2.34)	<.001
Lipid profile*						
TC	0.63 (0.50–0.79)	<.001	0.70 (0.55–0.88)	.003	0.64 (0.49–0.83)	<.001
TG	0.67 (0.60–0.75)	<.001	0.77 (0.68–0.87)	<.001	0.92 (0.8–1.07)	.284
LDL‐C < 1.89 mmol/L						
1.89 mmol/L ≤ LDL‐C < 2.41 mmol/L	0.77 (0.68–0.87)	<.001	0.79 (0.7–0.9)	<.001	0.86 (0.74–0.99)	.036
2.41 mmol/L ≤ LDL‐C < 3.03 mmol/L	0.80 (0.67–0.94)	.009	0.9 (0.75–1.07)	.243	0.95 (0.78–1.17)	.655
LDL‐C ≥ 3.03 mmol/L	0.68 (0.54–0.86)	.002	0.78 (0.61–1)	.053	0.67 (0.5–0.89)	.007
HDL‐C	0.79 (0.6–1.03)	.08	0.65 (0.49–0.86)	.003	0.77 (0.55–1.07)	.119

*Note*: Model 1 indicated unadjusted. Model 2 was adjusted for age, sex, and baseline body mass index (BMI). Model 3 was adjusted for systolic blood pressure, heart rate, serum sodium, total bilirubin, alanine transaminase (ALT), total cholesterol, creatinine, lg NT‐proBNP, left ventricular ejection fraction (LVEF), New York Heart Association (NYHA), ischemic cause of heart failure, medication of β‐blocker, angiotensin concerting enzyme inhibitor (ACEI), angiotensin receptor antagonist (ARB), aldosterone receptor antagonist (MRA), comorbidities of hypertension, diabetes, or atrial fibrillation.

Abbreviations: ACEI, angiotensin‐converting enzyme inhibitor; ALT, aspartate transaminase; ARB, angiotensin receptor blocker; BMI, body mass index; cTnI, cardiac troponin I; HbAlc, glycosylated hemoglobin; HDL‐C, high‐density lipoprotein cholesterol; hsCRP, high‐sensitivity C‐reactive protein; LDL‐C, low density‐lipoprotein cholesterol; LVDD, left ventricular end‐diastolic dimension; LVEF, left ventricular ejection fraction; NT‐proBNP, N‐terminal pro‐B‐type natriuretic peptides; NYHA, New York Heart Association; TBiL, total bilirubin; TC, total cholesterol; TG, triglyceride.

* Lipid profile were adjusted for Model 3, excluding total cholesterol.

### 3.3 Survival analysis based on lipid profile

In the analysis of the lipid profile shown in Table 2, higher TC (HR 0.64; 95% CI: 0.49–0.83; *p* < .001) was a protective factor in HF patients even after adjustment for the maximum number of variables, see Model 3. We observed a notable association between LDL‐C and composite outcome; patients with LDL‐C values in Q2 (1.89 mmol/L ≤ LDL‐C < 2.41 mmol/L), and Q4 (LDL‐C ≥ 3.03 mmol/L) had 0.86 (95%CI: 0.74–0.99; *p* = .036) and 0.67 (95%CI:0.5–0.89; *p* = .007) lower risk compared with patients in Q1(LDL‐C < 1.89 mmol/L), respectively. Higher TG seemed to be a protective factor in HF patients both in univariate (HR 0.67; 95%CI: 0.60–0.75; *p* < .001) and multivariate Cox regression analysis (HR 0.77; 95%CI: 0.68–0.87; *p* < .001) adjusted for Model 2, however not significant in Model 3 (*p* = .284). Figure S1 indicated that lower TC, TG, LDL‐C, and HDL‐C, divided into subgroups according to median or quartiles values, showed negative outcomes in Kaplan‐Meier survival curves (*p* < .001).

### 3.4 Relationship between thyroid hormone levels and lipid profile

**Figure 3:**
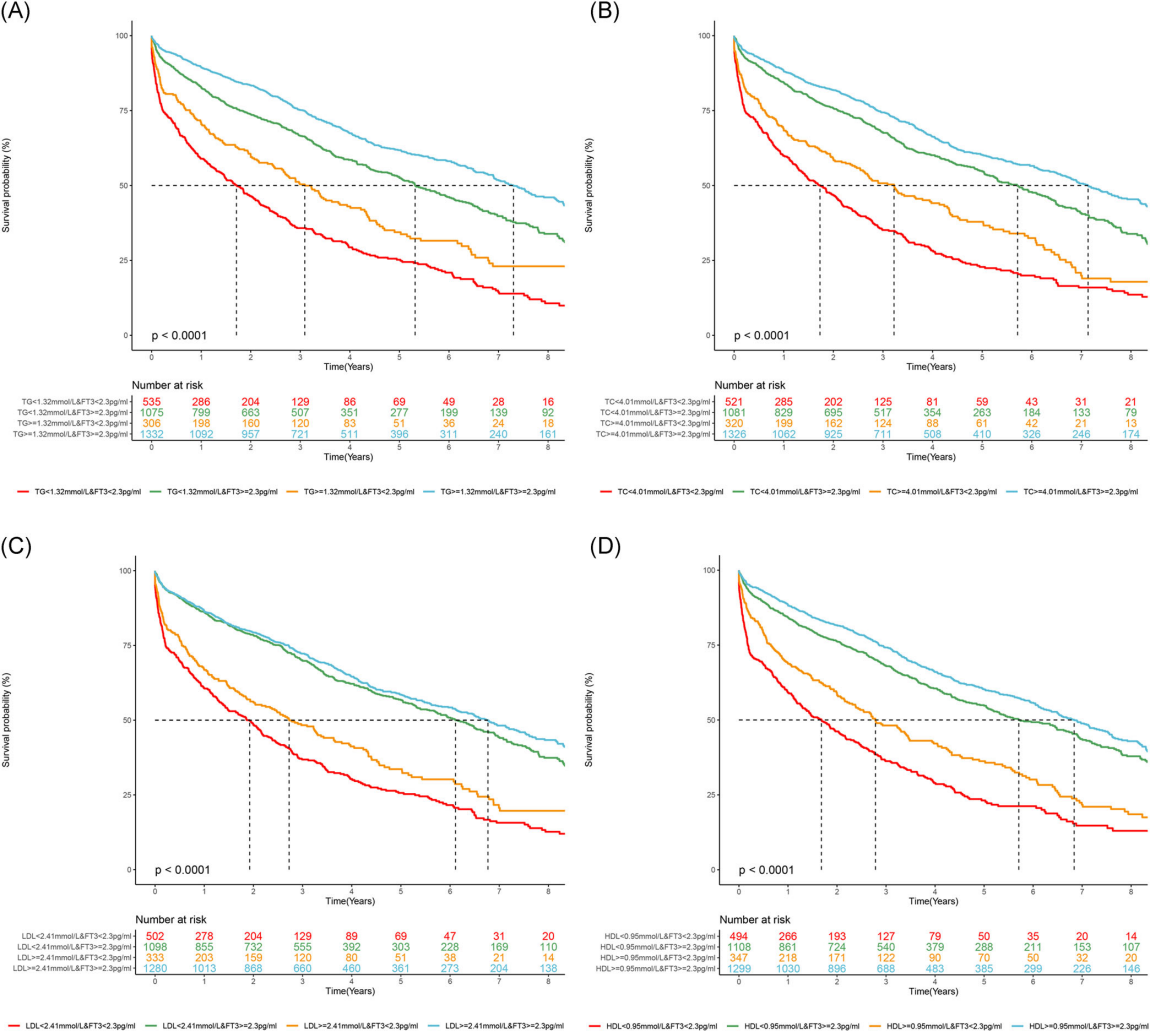
Kaplan–Meier analysis for composite endpoint of patients subdivided into four groups based on lower limit of normal for fT3 (2.3 pg/mL) and median value of lipid profile.

Analysis regarding the association between thyroid hormones and the parameters of lipid profile was shown in Table S1. fT3 is positively related with TC (*ρ* = .195, *p* < .001), TG (*ρ* = .238, *p* < .001), HDL‐C (*ρ* = .144, *p* < .001) and LDL‐C (*ρ* = .168, *p* < .001). Significant positive relationships were still detected between TT3, TT4, and the lipid profile mentioned above (*p* < .001). As shown in Figure 3, when patients were subdivided based on a lower limit of normal for fT3 (2.3 pg/mL) and median TC (4.01 mmol/L), lower fT3 combined with lower TC showed negative outcome in Kaplan–Meier survival curves (*p* < .001). Similar results were observed in fT3 combined with TG, LDL‐C, and HDL‐C (*p* < .001).

## 4 DISCUSSION

As reported in the present study, LT3S, overt hyperthyroidism, subclinical hypothyroidism, and overt hypothyroidism were independently associated with composite endpoints including all‐cause mortality, heart transplantation, or left ventricular assist device requirement in hospitalized HF patients. Lower fT3 and elevated TSH were independent prognosticators of worse outcomes in HF. Moreover, fT3 was positively related to lipid profiles, and fT3 combined with lipid profile improved risk stratification of the composite endpoint.

Thyroxine (T4) and triiodothyronine (T3), produced peripherally by T4 5′‐monodeiodination, have multiple effects on cardiovascular function, mediated by different signaling pathways that have been clustered into genomic and non‐genomic action. Thyroid hormones bind to thyroid hormone receptor‐α and β in cardiac myocytes to regulate gene expression.^12^ In addition, thyroid hormones exert nongenomic effects via directly binding to specific targets to mediate thyroid hormone action on the transport of ions across the plasma membrane and glucose and amino acid transport.^13^ Therefore, thyroid hormones play a crucial role in the cardiovascular system, including HF.

Both overt and subclinical hypothyroidism are associated with several cardiovascular risk factors such as dyslipidemia, hypertension, accelerated atherosclerosis, and coronary artery disease,^14^ the previous studies aimed to examine the prognostic role of low thyroid hormones in patients hospitalized for HF have produced conflicting results. A meta‐analysis of 21 221 patients with HF from 14 studies demonstrated that subclinical hypothyroidism could increase the risk of both all‐cause mortality and cardiac death and/or hospitality in HF patients.^2^ Selmer et al.^15^ found reduced all‐cause mortality in subclinical hypothyroidism (TSH levels of 5–10 mLU/L) with no increase in major adverse cardiovascular events, while no effect on all‐cause mortality or cardiovascular events of overt hypothyroidism was observed. A possible explanation of the inconsistent results is the heterogeneity of patients enrolled in terms of HF cause, HF status, sex, age, the level of TSH, and time of thyroid hormone sampling in different studies. In the present study, both overt and subclinical hypothyroidism had independently associated with all‐cause mortality in HF at univariate as well as multivariate analysis, which was in line with a previous study enrolled acute HF with similar baseline characteristics,^16^ and this finding was consistent with the recommendations by European Society of Cardiology (ESC) to monitor thyroid hormone levels^17^ and to initiate treatment early in case of overt hypothyroidism. However, the negative effects of subclinical hyperthyroidism were not seen in our study, like previous studies.^18^, ^19^ A possible explanation is that the lower prevalence and the potential positive effects, such as increased contractility and reduced systemic vascular resistance, mitigated the negative effects.^20^ Although the negative prognostic effect of overt hyperthyroidism was observed in our study, in consideration of the relatively small samples, further studies with a larger cohort of overt hyperthyroidism in HF patients are urgently needed.

An increased TSH, indicating a deficiency of circulating thyroid hormones, was independently associated with a higher incidence of composite outcomes, including death, urgent heart transplantation, or hospitalization because of worsening HF.^21^ In the extremely severe stage of HF, peripheral T4–T3 conversion would be more strongly suppressed and subsequently increase the secretion of TSH via feedback of the hypothalamic‐pituitary‐thyroid axis. Therefore, elevated TSH and low fT3 on the admission of acute decompensated HF patients might indicate the severe or life‐threatening systemic state in the HF phase.^19^ In our series, we demonstrated that both elevated TSH and low fT3 levels on admission were independent adverse markers in HF patients requiring intensive care. Moreover, though LT3S was traditionally considered an adaptive compensatory response in severe clinical conditions by reducing body energy consumption, it was reported to be associated with increased length of hospitalization, higher rate of intense care unit admission,^5^ higher cardiac mortality, and all‐cause mortality.^22^ However, such associations were not observed in another single‐center study, which may be explained by the heterogeneity in sampling timing and the different phase of HF in the study population.^19^

Thyroid hormones, even within the normal ranges, may contribute to the alternation in lipid metabolism. Hypothyroidism and subclinical hypothyroidism have significantly elevated levels of TC, TG, and LDL‐C^23^, ^24^ in the general population. It might be the explanation that the combination of T3 and the sterol regulatory element binding proteins (SREBP) upregulate the synthesis of LDL‐C receptors along with the regulatory enzymes of cholesterol synthesis. Therefore the increase of T3 leads to an increase in LDL‐C levels through the reduction of LDL‐C receptors.^25^ However, HF patients with overt hypothyroidism and LT3S, who have worse outcomes, were associated with lower lipid profiles in our cohort, and the correlation analysis showed a positive trend between thyroid hormones and comprehensive parameters of lipid profile. Several studies revealed higher mortality in HF with lower total cholesterol levels,^26^ which was the so‐called reverse epidemiology. Thus, the traditional risk factors of poor clinical outcomes in the general population, such as higher cholesterol, BMI, and systolic blood pressure paradoxically associated with better survival in HF.^26^ Our study showed that the combination of dyslipidemia and fT3 could significantly improve the outcome risk stratification. However, the mechanisms underlying this association still need to be elucidated. There were a number of plausible hypotheses that may tentatively explain that such as the gastrointestinal congestion was partly associated with the development of low T3 syndrome through appetite loss and overall reduction in intestinal absorption of dietary lipids, which were also associated with malnutrition and cardiac cachexia in HF.^22^

Though we explored the prognostic value of thyroid dysfunction in patients with pre‐existing HF requiring intense care and evaluated the association between the lipid profile and thyroid hormones in HF patients for the first time. Some limitations could not be neglected. First, it is a single‐center retrospective study, the possibility of residual confounding cannot be excluded and need to be validated in larger cohort studies. Secondly, thyroid function was assessed only once at baseline. We were unable to assess the prognostic value of transient changes in thyroid function in HF patients. Thirdly, there was heterogeneity in the cause of HF, and the medication after discharge was unavailable.

## 5 CONCLUSION

The presence of LT3S, overt hyperthyroidism, and overt and subclinical hypothyroidism on admission for HF requiring intense care were independent poor prognostic indicators. Low fT3 and elevated TSH could serve as promising markers to predict adverse outcomes in HF patients. Low fT3 seemed to be associated with a lower level of lipid profiles in HF, and the combination of fT3 and parameters of lipid profile improves the power of risk stratification.

## AUTHOR CONTRIBUTIONS

Yuhui Zhang and Jian Zhang designed and supervised the study. Ping Zhou, Liyan Huang, Mei Zhai, Yan Huang, Xiaofeng Zhuang, and Huihui Liu performed sample and data acquisition. Ping Zhou and Liyan Huang performed data analysis and interpretation. Ping Zhou wrote the manuscript. Yuhui Zhang and Jian Zhang approved manuscript submission. All authors read and contributed to the manuscript.

## CONFLICT OF INTEREST STATEMENT

The authors have no potential conflict of interest to declare.

## Supporting information

Figure S1. Kaplan–Meier analysis for composite endpoint on the basis of lipid profile in heart failure.

Supplementary information.

## Data Availability

The data that support the findings of this study are available on request from the corresponding author. The data are not publicly available due to privacy or ethical restrictions.
