# Supplementary figures and images for: Prognostic role and relationship of thyroid dysfunction and lipid profile in hospitalized heart failure patients
Source: Clin Cardiol. 2023 May 25;46(7):757–67. doi: 10.1002/clc.24057 (PMC10352970; doi:10.1002/clc.24057)

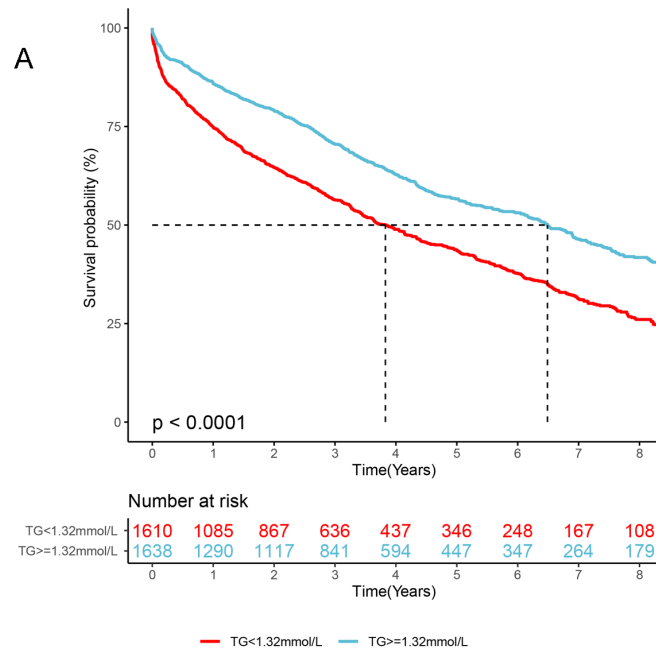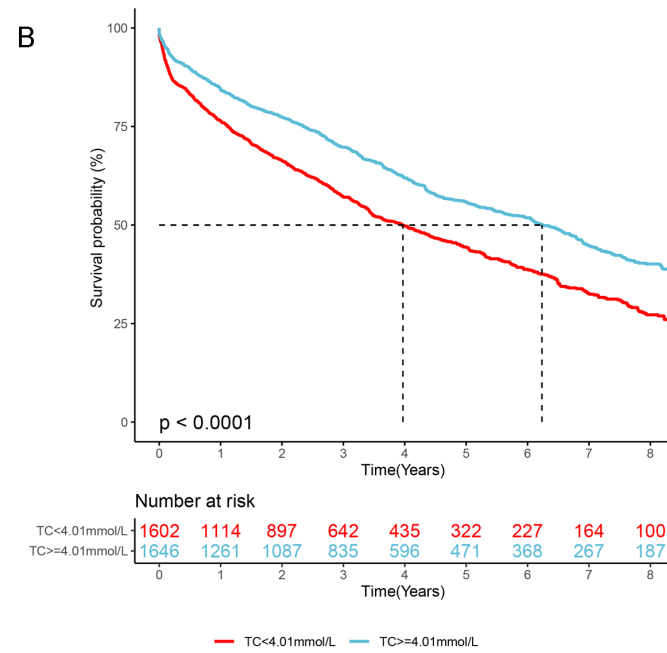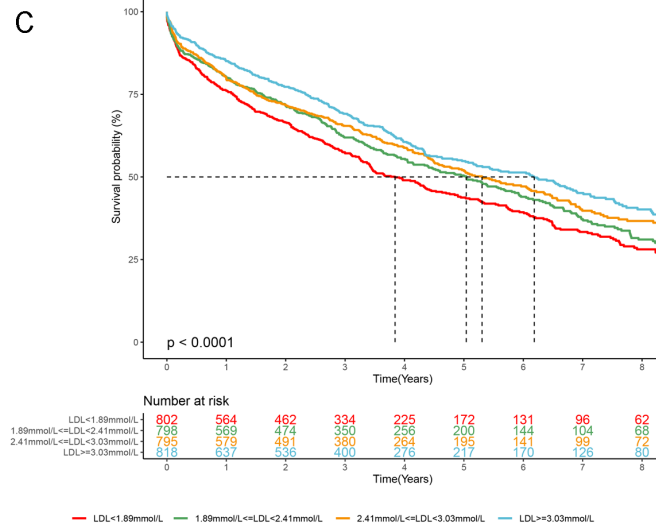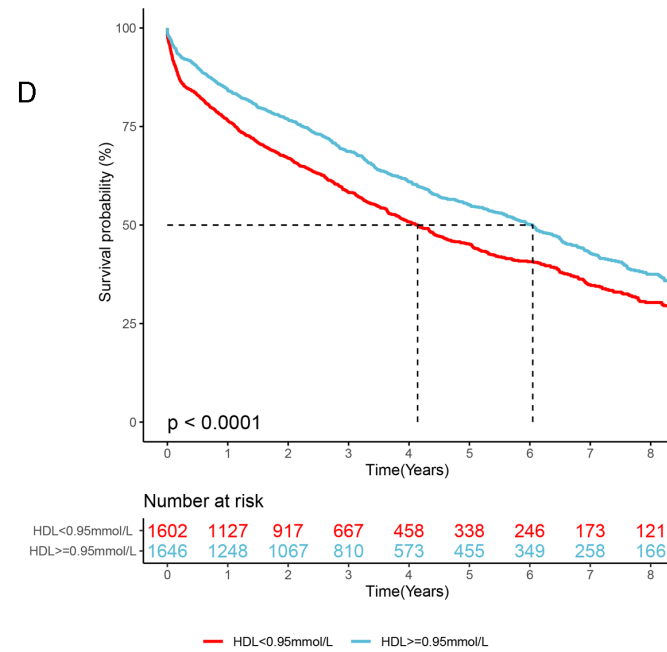

Supplement: Supplementary file 1 — Figure S1. Kaplan–Meier analysis for composite endpoint on the basis of lipid profile in heart failure. [file CLC-46-757-s001.pdf]
