# Supplementary material for: Prognostic role and relationship of thyroid dysfunction and lipid profile in hospitalized heart failure patients
Source: Clin Cardiol. 2023 May 25;46(7):757–67. doi: 10.1002/clc.24057 (PMC10352970; doi:10.1002/clc.24057)
Supplement: Supplementary file 2 — Supplementary information. [file CLC-46-757-s002.docx]

**Table S1. Correlations between lipid profile and thyroid functions in patients with heart failure.**

|  | **fT3** | | **fT4** | | **TSH** | | **TT3** | | **TT4** | |
| --- | --- | --- | --- | --- | --- | --- | --- | --- | --- | --- |
|  | Sperman rho | P value | Sperman rho | P value | Sperman rho | P value | Sperman rho | P value | Sperman rho | P value |
| TC | 0.195 | <0.001 | 0.058 | <0.001 | -0.007 | 0.653 | 0.278 | <0.001 | 0.148 | <0.001 |
| TG | 0.238 | <0.001 | 0.021 | 0.197 | 0.028 | 0.086 | 0.322 | <0.001 | 0.119 | <0.001 |
| HDL-C | 0.144 | <0.001 | -0.002 | 0.889 | 0.031 | 0.062 | 0.161 | <0.001 | 0.069 | <0.001 |
| LDL-C | 0.168 | <0.001 | 0.081 | <0.001 | -0.008 | 0.648 | 0.162 | <0.001 | 0.122 | <0.001 |

Abbreviation: fT3, free triiodothyronine; fT4, free thyroxine; TT3, total triiodothyronine; TT4, total thyroxine; TSH, thyroid-stimulating hormone; TC, total cholesterol; TG, triglyceride; LDL-C, low density-lipoprotein cholesterol; HDL-C,high density-lipoprotein cholesterol;
